# Prevalence and risk factors of stroke in the elderly in Northern China: data from the National Stroke Screening Survey

**DOI:** 10.1007/s00415-019-09281-5

**Published:** 2019-04-15

**Authors:** Xiaoshuang Xia, Wei Yue, Baohua Chao, Mei Li, Lei Cao, Lin Wang, Ying Shen, Xin Li

**Affiliations:** 1 Department of Neurology, The Second Hospital of Tianjin Medical University, Tianjin, 300211 China; 2 Department of Neurology, Tianjin Huanhu Hospital, Tianjin, China; 3 Nation Project Office of Stroke Prevention and Control, Beijing, China; 4 China Stroke Data Center, Beijing, China; 5 Department of Geratology, The Second Hospital of Tianjin Medical University and Tianjin Geriatric Institute, Tianjin, China; 6 Department of Traditional Chinese Medicine, Beijing Xuanwu Hospital, Capital Medical University, Beijing, 100000 China

**Keywords:** Prevalence, Risk factors, Stroke, Elderly

## Abstract

**Background:**

The overall global burden of stroke is considerable and increasing. In China, stroke is the leading cause of death and disability.

**Methods:**

For this study, we used data from the National Stroke Screening Survey in 2012 and the 2010 Chinese population from sixth National Census of Populations to calculate a standardized (by age, gender, and education) stroke prevalence. Prevalence, risk factors, and management of stroke were compared by gender, age, and site.

**Findings:**

The standardized prevalence rate of survival stroke patients in study population aged 60 and older was 4.94% in total. Hypertension was the most prevalent risk factor for stroke. Compared to men, women were more likely to have diabetes, obesity, elevated low-density lipoprotein cholesterol (LDL-C), and atrial fibrillation (*P* < 0.05). Men were far more likely to drink and smoke than women (*P* < 0.05). The rates of diabetes and atrial fibrillation were substantially higher in urban than those in rural stroke survivors (*P* < 0.05). Rural stroke survivors exhibited higher rates of smoking and alcohol consumption than urban stroke survivors (*P* < 0.05).

**Interpretation:**

The stroke prevalence in China is in line with median worldwide stroke prevalence. Traditional risk factors remain highly prevalent in stroke survivors, among which hypertension was the most common. Stroke prevalence rates and risk factors varied by age, sex, and sociogeological factors.

**Electronic supplementary material:**

The online version of this article (10.1007/s00415-019-09281-5) contains supplementary material, which is available to authorized users.

## Introduction

Stroke is the second most frequent cause of death and a leading cause of disability worldwide [1]. Although age-standardized rates of stroke mortality have decreased worldwide in the past two decades, the absolute numbers of people who have a stroke each year, stroke survivors, and stroke-related deaths as well as the overall global stroke burden are considerable and increasing [2]. The stroke burden is particularly serious in Asia [3], where its mortality rate is higher than in Europe or North America [4]. In China, stroke is the leading cause of death and disability [5]. According to the Sixth National Census of Population (6th NCP) in 2010 in China, the proportion of those aged 60 years and older accounted for 13.26% of the population, representing a 2.93% increase over the prior 10 years. Population ageing has driven up the disease burden and, as a result, the stroke burden in particular has arisen to the leading disease burden in China [6]. The risk of stroke rises markedly with advancing age due to multiple risk factors and is associated with poorer outcomes [7].

Stroke prevalence also varies geographically in China [5]. Previous studies had shown a stroke belt that includes Heilongjiang, Tibet, Jilin, Liaoning, Xinjiang, Hebei, Inner Mongolia, and Beijing [8]; eight of these nine provinces belong to the northern region. The incidence of stroke was reported to be higher in the north, with the highest incidence recorded in Huabei and the north-east of China; the lowest incidence was recorded in the south [9]. According to the 2010 China Census, the national population (excluding Taiwan, Hong Kong, and Macao) was 1.339 billion, of whom 560 million (41.8%) resided in Northern China. The northern area covers approximately 60% of Chinese territory. Thus, it is important to understand the prevalence and risk factors of stroke in Northern China. As the data were limited in previous studies, this study aims to provide new insights into the prevalence, risk factors, prevention, and management strategies of stroke among the elderly in Northern China.

## Methods

In China, the National Stroke Screening Survey was launched by the Chinese Ministry of Health in 2011 with the commitment to prevent and manage stroke. The screening program was implemented in two stages. In the first stage, using a 2-stage stratified cluster sampling method, communities were selected in proportion to the local population size. The cluster sampling method was used in every primary sampling unit, and all residents aged 40 years or older in the selected communities were invited to participate in the first stage of the screening program, which was conducted in nearby community hospitals or health stations. The participants were invited via telephone. Trained researchers interviewed participants face to face regarding stroke history and risk factors as well as demographic information (age, gender, education level, etc.). In the second stage, participants who had a history of stroke were interviewed again for additional details about their stroke histories and risk factors, including information on smoking, alcohol consumption, physical activity, hypertension, diabetes. Physical and laboratory examinations (serum lipids and glucose) were also performed. Only researchers who had been trained on the standardized process and passed a training examination were able to participate and professional quality control personnel supervised the conduction of the study.

For this study, we used the data collected from those aged 60 years and older in Northern China (Beijing, Hebei, Shanxi, Liaoning, Heilongjiang, Shandong, Henan, Shaanxi, Gansu, Tianjin, Xinjiang, Jilin, Ningxia, Inner Mongolia) in 2012. Places of residence were classified as urban or rural by address.

We recruited 144,722 participants aged 60 years and older through population-based sampling from 14 provinces in the first stage of the screening program, according to the data from the 6th NCP. Supplemental Table 1 shows characteristics of the patient population including the distribution of gender, age, geographic region, and urban or rural residence. Women constituted 54.79% of the patient population, and 46.35% of the study population were urban. The population aged 60–69 years comprised 60.88% of the study population, while the very elderly (≥ 80 years old) comprised 8.93%. These data were similar to those of the 6th NCP.

### Study methods

We focused on analyzing major risk factors including hypertension, diabetes, hyperlipidemia, and atrial fibrillation prior to stroke. Hypertension was defined as systolic blood pressure ≥ 140 mmHg or diastolic blood pressure ≥ 90 mmHg after repeated measurement or a history of antihypertensive agent use. Diabetes was defined as a self-reported history of diabetes. Dyslipidemia was defined as a self-reported physician diagnosis or as abnormal fasting (> 8 h) levels of any of the following: serum total cholesterol, triglycerides, or low-density lipoprotein. Atrial fibrillation was defined as any electrocardiographic evidence of atrial fibrillation or self-reported physician diagnosis. Body mass index (BMI) was calculated as weight in kilograms divided by the square of height in meters and overweight or obese was defined as BMI ≥ 26 kg/m^2^. Physical activity less than 3 times a week for 30 min was considered physical inactivity. Prior stroke history, including ischemic stroke (IS) or intracerebral hemorrhage stroke (ICH), was defined as clinical presentation with confirmation by computed tomography (CT) or magnetic resonance imaging (MRI) as diagnosed by a neurologist based on clinical records. In patients with a previous stroke, the Essen Stroke Risk Score (ESRS) was used to stratify the risk of recurrent stroke or major vascular event [10]. The institutional ethics committee approved the study protocol.

### Statistical analysis

Categorical variables are displayed as frequencies and percentages. Categorical variables were analyzed using a Chi-square (*χ*^2^) test. We calculated standardized stroke prevalence (by age, sex, and education) with data from the 6th NCP as standards. The Poisson model was used to determine 95% confidence intervals (CI). A two-tailed *P* < 0.05 was considered statistically significant.

## Results

### Stroke prevalence among the elderly in Northern China

**Fig. 1:**
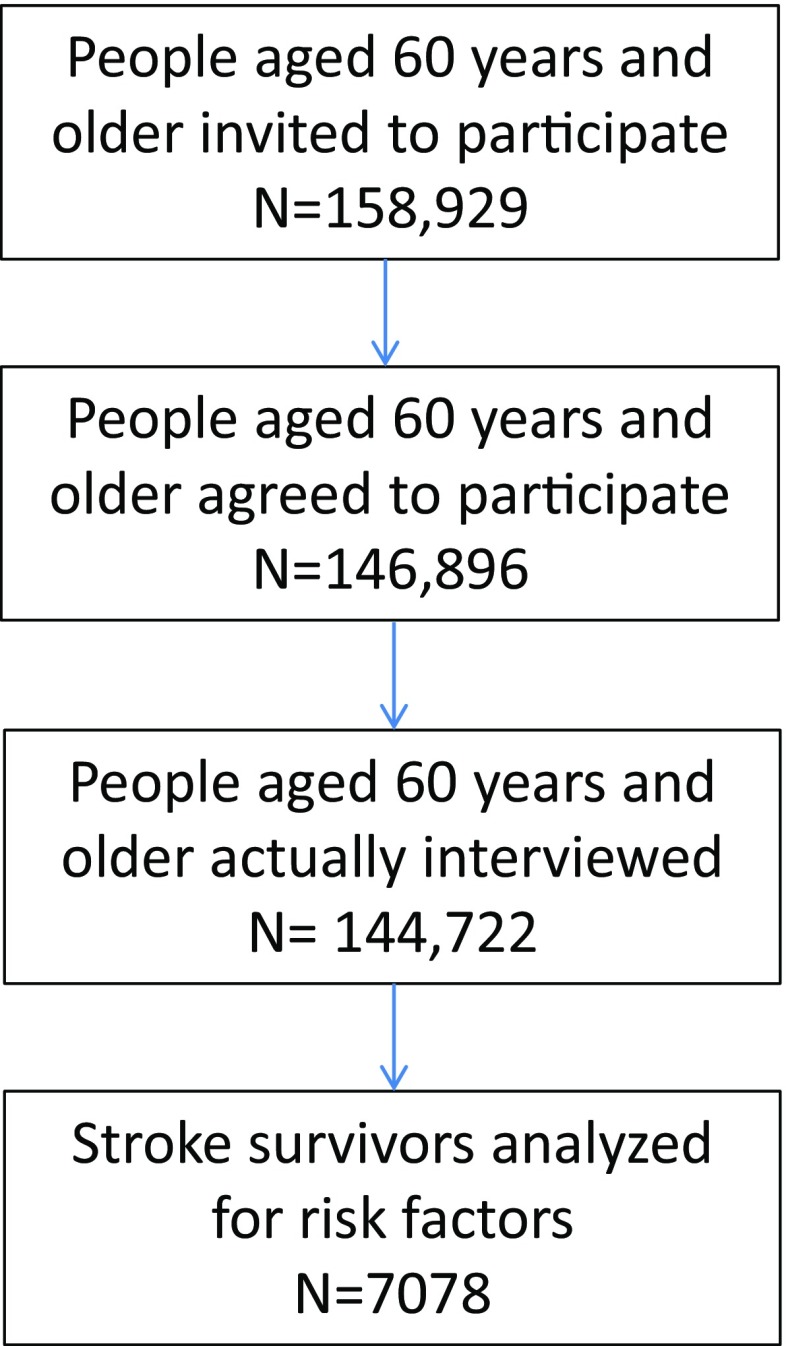
Flowchart of subjects recruited into the study

**Fig. 2:**
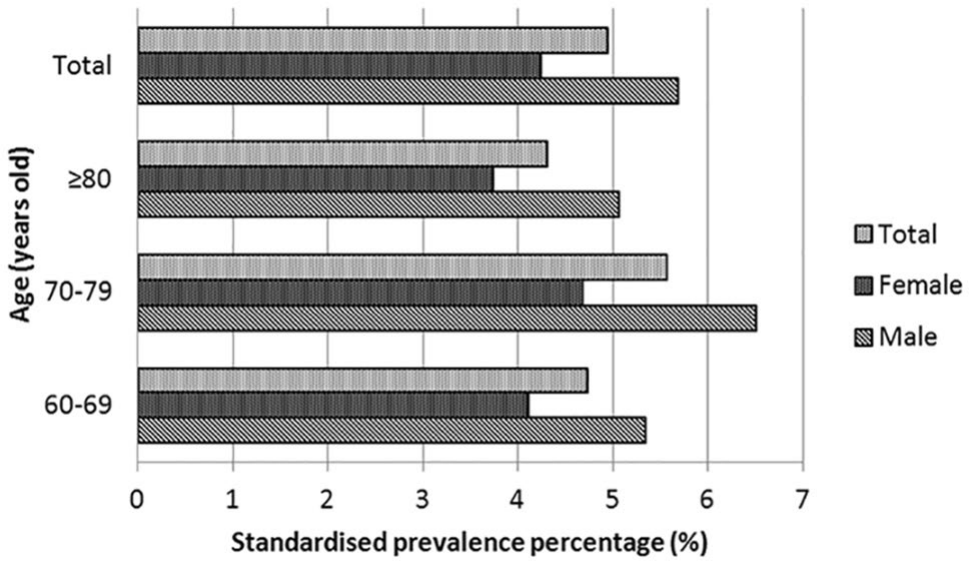
Stroke prevalence among patients aged 60 years and older in Northern China. We calculated the standardized stroke prevalence (for age, sex, and education) using the 2010 Chinese population from sixth National Census of Population

158,929 residents aged 60 years and older were invited to participate in the study, and 146,896 residents agreed to take part in the study. At last, 144,772 participants aged 60 years and older were actually interviewed (Fig. 1). Screening identified 7,078 stroke survivors among the study population of 144,722. The crude stroke prevalence rates in the study population aged 60 years and older were 4.89% in total, 5.67% in men, and 4.25% in women. The stroke prevalence rate in the entire study population was 4.94% after standardizing for gender, age, educational level, urban or rural residence, and geographic region using the 6th NCP. The stroke prevalence rate was highest among population aged 70–79 years (5.56%) (Fig. 2). Men had a higher standardized stroke prevalence rate than women (5.68% vs. 4.24%). Stroke prevalence was slightly higher in rural areas than in urban areas (5.04% vs. 4.82%; Table 1).

**Table 1:** Stroke prevalence rate of population aged 60 years and older in Northern China

	Male			Female			Total		
	*n*	Crude prevalence percentage (95% CI)	Standardized^a^ prevalence percentage (95% CI)	*n*	Crude prevalence percentage (95% CI)	Standardized^a^ prevalence percentage (95% CI)	*n*	Crude prevalence percentage (95% CI)	Standardized^a^ prevalence percentage (95% CI)
Total	3709	5.67 (5.53–5.81)	5.68 (5.50–5.86)	3369	4.25 (4.11–4.39)	4.24 (4.10–4.39)	4.89	4.89 (4.78–5.00)	4.94 (4.83–5.06)
Age									
60–69	2117	5.34 (5.12–5.56)	5.34 (5.11–5.57)	1996	4.12 (3.94–4.29)	4.11 (3.93–4.29)	4113	4.67 (4.53–4.81)	4.72 (4.58–4.87)
70–79	1294	6.50 (6.15–6.84)	6.50 (6.14–6.85)	1108	4.66 (4.39–4.93)	4.67 (4.39–4.94)	2402	5.50 (5.28–5.71)	5.56 (5.33–5.78)
≥ 80	298	5.06 (4.50–5.62)	5.06 (4.48–5.63)	265	3.76 (3.32–4.21)	3.73 (3.28–4.18)	563	4.36 (4.00-4.71)	4.30 (3.95–4.66)
Provinces									
Beijing	294	5.65 (5.17–6.14)	7.25 (4.57–9.92)	314	4.45 (3.97–4.93)	5.77 (3.64–7.91)	608	4.96 (4.58–5.35)	6.49 (4.79–8.19)
Tianjin	255	5.54 (5.00-6.08)	5.50 (4.82–6.18)	242	4.38 (3.84–4.92)	4.33 (3.77–4.88)	497	4.91 (4.49–5.33)	4.90 (4.46–5.34)
Hebei	348	7.57 (7.03–8.11)	7.16 (6.18–8.13)	286	4.79 (4.25–5.34)	4.61 (3.86–5.35)	634	6.00 (5.55–6.46)	5.85 (5.24–6.46)
Shanxi	222	4.28 (3.86–4.70)	4.19 (3.64–4.75)	160	2.77 (2.34–3.19)	2.72 (2.29–3.15)	382	3.48 (3.14–3.82)	3.44 (3.09–3.79)
Inner Mongolia	111	6.61 (5.83–7.39)	6.62 (4.40–8.84)	101	4.06 (3.29–4.84)	3.66 (2.37–4.96)	212	5.09 (4.42–5.76)	5.10 (3.83–6.37)
Liaoning	264	6.73 (6.03–7.43)	6.06 (5.21–6.92)	257	5.89 (5.19–6.59)	6.01 (5.13–6.89)	521	6.29 (5.76–6.81)	6.04 (5.42–6.65)
Jilin	171	6.86 (6.00-7.72)	7.13 (6.01–8.25)	176	6.01 (5.15–6.87)	6.63 (5.52–7.73)	347	6.40 (5.75–7.05)	6.87 (6.09–7.66)
Heilongjiang	129	6.09 (5.04–7.14)	5.91 (4.54–7.28)	158	6.98 (5.93–8.03)	8.30 (6.40–10.20)	287	6.55 (5.82–7.28)	7.14 (5.96–8.32)
Shandong	894	6.20 (5.89–6.50)	6.47 (5.98–6.95)	795	4.52 (4.21–4.82)	4.64 (4.27–5.01)	1689	5.27 (5.03–5.52)	5.53 (5.22–5.83)
Henan	500	5.67 (5.33–6.02)	5.60 (5.10–6.10)	439	3.75 (3.40–4.09)	3.68 (3.32–4.04)	939	4.57 (4.29–4.86)	4.61 (4.31–4.92)
Shanxi	217	4.58 (4.07–5.09)	3.59 (2.92–4.25)	153	3.26 (2.75–3.77)	2.67 (2.13–3.22)	370	3.92 (3.53–4.32)	3.12 (2.69–3.54)
Gansu	153	4.06 (3.60–4.53)	4.28 (3.49–5.07)	104	2.46 (1.99–2.93)	2.57 (1.98–3.17)	257	3.22 (2.83–3.60)	3.41 (2.91–3.90)
Ningxia	78	5.75 (4.41–7.08)	6.05 (4.57–7.53)	99	7.03 (5.70–8.37)	6.53 (4.92–8.14)	177	6.40 (5.49–7.31)	6.30 (5.20–7.39)
Xinjiang	73	2.89 (2.34–3.43)	2.96 (2.04–3.88)	85	2.60 (2.05–3.14)	2.44 (1.79–3.10)	158	2.72 (2.30–3.14)	2.70 (2.14–3.25)
Residents									
Urban	1655	5.54 (5.28–5.80)	5.55 (5.28–5.82)	1539	4.13 (3.93–4.34)	4.15 (3.94–4.36)	3194	4.76 (4.60–4.92)	4.82 (4.66–4.99)
Rural	2054	5.77 (5.53–6.01)	5.79 (5.54–6.04)	1830	4.35 (4.16–4.55)	4.32 (4.12–4.52)	3884	5.00 (4.85–5.16)	5.04 (4.88–5.20)

^a^Standardized for age, sex, and education

The standardized stroke prevalence rate varied considerably among provincial regions, ranging from 2.7% in Xinjiang to 7.14% in Heilongjiang; there was nearly a three-fold difference in stroke prevalence between provinces with the highest and the lowest stroke prevalence. According to the geographic positions of the provinces, stroke prevalence was higher in northeast China (Heilongjiang, Jilin, and Liaoning) than in other areas, with prevalence rates above 6% in each northeast province. Other provinces lying in east or east-central China (Beijing, Tianjin, Shandong, Hebei, Inner Mongolia, Shanxi and Henan) had higher stroke prevalence than those in the west (Shaanxi, Gansu, and Xinjiang). One exception is Ningxia, located in northwest China, which had a higher prevalence rate than other western provinces. (Supplemental Fig. 1).

When classified by socioeconomic level, high-income (Beijing, Tianjin, Shandong, Liaoning, and Inner Mongolia) and middle-income provinces (Hebei, Shanxi, Jilin, and Heilongjiang) had higher stroke prevalence rates than low-income provinces (Gansu, Henan, Xinjiang, and Shaanxi; *P* < 0.05). Again, the low-income province Ningxia, with a high stroke prevalence rate, represents an exception.

### Recurrent stroke risk stratification in stroke survivors

According to ERS, 68.89% patients were identified as high risk for recurrent stroke. The proportion of high-risk patients among men was higher than among women (73.39% vs. 63.94%; *P* < 0.05). Nearly, 80% of stroke survivors aged 70 years and older were stratified into the high risk group, which was significantly higher than the proportion patients aged under 70 years who were at high risk for recurrent stroke (*P* < 0.05; Supplemental Table 2).

### Risk factors in stroke survivors

**Fig. 3:**
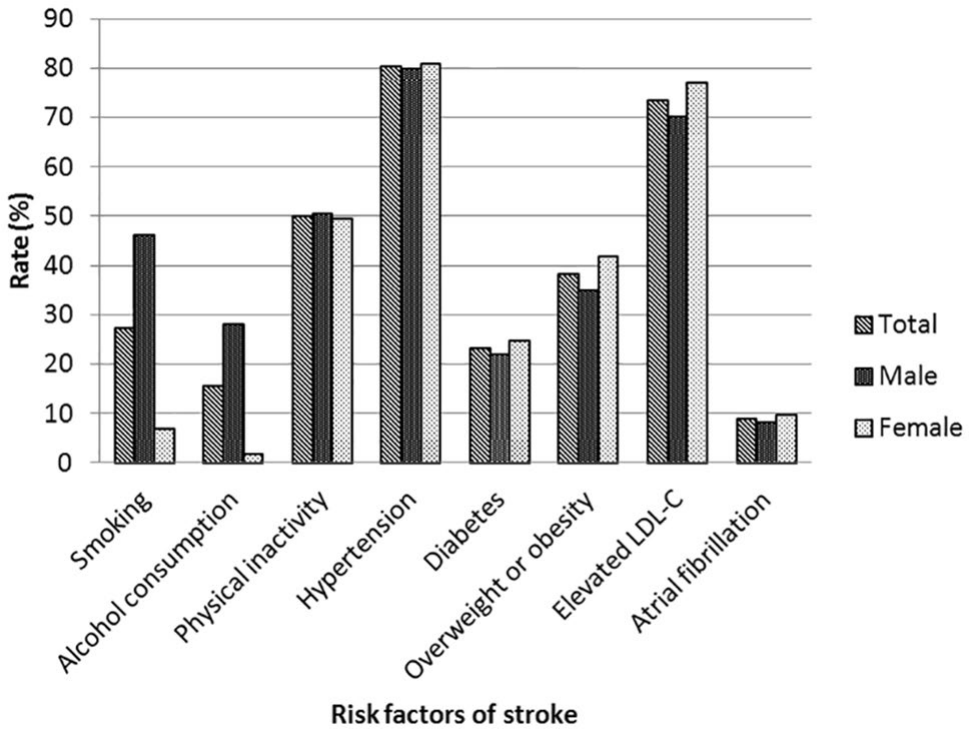
Main risk factors for stroke in stroke survivors. Hypertension was the most prevalent risk factor for stroke. Compared with men, women were more likely to have diabetes, obesity, elevated LDL-C, and atrial fibrillation (*P* < 0.05). Men were far more likely than women to drink and smoke (*P* < 0.05). No significant gender difference was detected in hypertension or physical inactivity (*P* > 0.05)

Of the stroke survivors, 80% had hypertension, which was the most prevalent risk factor for stroke. Compared with men, women were more likely to have diabetes, obesity, elevated LDL-C, and atrial fibrillation (*P* < 0.05). Men were more far more likely to drink and smoke than women (*P* < 0.05). No significant gender differences were detected in hypertension or physical inactivity (*P* > 0.05). Risk factors varied by age, which is also shown in Table 2. The rates of smoking, alcohol consumption, diabetes, overweight or obese patients, and elevated LDL-C among stroke survivors aged 60–69 were higher than those among stroke survivors aged 70 or older (*P* < 0.05). No differences were detected in physical inactivity, hypertension, or atrial fibrillation among age subgroups (*P* > 0.05). The proportions of diabetes and atrial fibrillation were substantially higher among urban stroke survivors than rural stroke survivors (*P* < 0.05). Rural stroke survivors exhibited higher rates of smoking and alcohol consumption than urban stroke survivors (*P* < 0.05) (Fig. 3).

**Table 2:** Main risk factors of stroke in stroke survivors

	Smoking (%)	Alcohol consumption (%)	Physical inactivity (%)	Hypertension (%)	Diabetes (%)	Overweight or obese (%)	Elevated LDL-C (%)	Atrial fibrillation (%)
Total	27.26	15.45	50.01	80.18	23.24	38.14	73.36	8.80
Gender								
Male	46.07	27.96	50.42	79.72	21.96	34.85	70.04	8.09
Female	6.77	1.83	49.56	80.69	24.64	41.72	76.98	9.59
*P*	< 0.001	< 0.001	0.476	0.316	0.008	< 0.001	< 0.001	0.026
Age								
60–69	29.24	17.66	49.72	79.85	23.64	41.97	74.83	8.68
70–79	25.53	12.97	50.78	81.06	24.21	34.93	71.45	9.08
≥ 80	19.96	9.70	48.79	78.83	16.14	23.76	70.67	8.53
*P*	< 0.001	< 0.001	0.598	0.355	< 0.001	< 0.001	0.004	0.838
Residents								
Urban	24.80	14.51	50.72	79.33	26.76	38.51	73.66	10.11
Rural	29.49	16.31	49.36	80.93	20.28	37.82	73.11	7.72
*P*	0.002	0.037	0.254	0.096	< 0.001	0.553	0.603	< 0.001

Supplemental Table 3 shows each main stroke risk factor in the 14 provincial regions of northern China. The proportion of hypertension was highest in Henan (85.3%) followed by Hebei (84%), and lowest in Shaanxi (59.1%). The proportion of diabetes was highest in Inner Mongolia (42.0%), followed by Beijing (32.4%). Ningxia, which is located in northwest China, had the fourth highest stroke prevalence (6.3%), the highest prevalence of atrial fibrillation, the second highest prevalence of elevated LDL-C, and the third highest prevalence of physical inactivity and overweight or obese patients.

## Discussion

### Stroke prevalence

In the present study, the standardized stroke prevalence rate among patients aged 60 years and older was 4.94%. Nationwide data on stroke prevalence in the elderly are rather sparse in China, especially in recent years [11]. According to the data collected during the 1990s, the age-adjusted stroke prevalence in China varies from 0.26 to 0.72% for patients of all ages [11]. A cross-sectional survey with a sample size of approximately 2,000 in China showed a stroke prevalence of over 5% among people aged 65 years and older [12]. The age inclusion criterion of that survey was 5 years older than that in our study, which might be a reason for the slightly higher prevalence found in that study than in the present study. Stroke prevalence in China is lower than in the United States, as the age-adjusted stroke prevalence in population aged 65 years and older was 8.3% in a nationwide survey in United States [13]. Stroke prevalence is much higher in China as compared to Africa, which has a stroke prevalence of less than 3% among population aged 60 years and older [14]. Among more developed countries, the age-standardized stroke prevalence for people aged 65 years or older ranges from 4.61 to 7.33% [15].

Stroke prevalence rates increase with age [13, 16]. The German Health Interview and Examination Survey for Adults (DEGS1) [16] from 2008 to 2011 showed that stroke prevalence increases with age under 80 years, which is consistent with our data. A nearly 10-fold difference in stroke prevalence estimate was observed between persons aged ≥ 65 years (8.3%) and those aged 18–44 years (0.7%) in United States [13]. However, the very elderly had the lowest prevalence rate (4.3%) in our study. A higher mortality rate and shorter mean length of survival after stroke in the very elderly might explain this difference [17]. Sex differences in stroke prevalence have been previously reported [13, 14, 16], which are consistent with the lower stroke prevalence among women that was found in our study.

One prior study found higher stroke prevalence in urban (9.3%) than in rural China (1.2%) [12]. We also detected a difference in stroke prevalence between urban and rural populations; however, in contrast to the aforementioned study, we found a slightly lower stroke prevalence in urban areas (4.8%) than in rural areas (5%). This finding is consistent with those of a prior study performed in Brazil [18]. Additionally, the incidence of stroke in rural China had increased rapidly in recent years [19].

A review of stroke epidemiology in China [11] found a decreasing north–south gradient of stroke prevalence. In our study, standardized stroke prevalence varied considerably in different provincial regions. The highest prevalence was found in Heilongjiang, similar to that found in the Sino-MONICA project [8], which also found the highest stroke incidence in Heilongjiang. According to the geographic position of the provinces, stroke prevalence was higher in the northeast of China than in other areas. Other provinces that lie in east or east-central China had higher stroke prevalence than those in west China. When classified by socioeconomic level, high- and middle-income provinces had higher stroke prevalence rates than low-income provinces in the present study. Contrary to our results, a survey in Germany found that stroke prevalence was at its highest among people of low socioeconomic status [16]. A higher stroke incidence had been observed in low as compared to high socioeconomic groups [20]. The data of this study were used to explore the prevalence of stroke in the elderly, which may explain this phenomenon. Socioeconomic differences at older ages may diminish compared with younger ages; less viable individuals may have died sooner [21]. In the low-income provinces, life expectancy is shorter [22], age of stroke onset is lower [23], and mortality is higher [24]. On the other hand, the elderly in low-income provinces experienced a period of economic underdevelopment in China when they were young, and residents in low-income areas were less exposed to certain risk factors of stroke such as high-fat and high-sugar diets and obesity, and mainly engaged in physical labor [25], which may result in lower stroke prevalence. With the development of China’s economy and a growing general awareness of stroke, this trend is likely to change and more match those of developed countries.

### Recurrent stroke risk stratification

In our study, nearly 70% of stroke survivors had ESRS ≥ 3, indicating a high risk of stroke recurrence. Similar results were described in the REACH Registry [26], which further confirmed the accuracy of ESRS in recurrent risk stratification. We also found that male stroke survivors and stroke survivors aged 70 years and older had a higher risk of stroke recurrence. In the Northern Sweden MONICA stroke incidence registry [27], correlations between age, sex, and recurrent stroke were identified. Thus, elderly patients with a high ESRS should be considered for comprehensive and individual secondary prevention strategies.

### Risk factors

Hypertension has been documented as the most important risk factor for stroke in China [11]. In our study, 80% of stroke survivors had hypertension, which was the most prevalent risk factor for stroke. A cohort study showed that the risk ratios (RRs) of total stroke with hypertension among ethnic Asian patients were higher than among Caucasian patients [28]. The second most common risk factor in our study population was elevated LDL-C levels. One large study showed a 14% increase in the relative risk of ischemic stroke per 40 mg/dL increase in LDL-C [29].

When biological sex was considered, this study found that women were more likely to have diabetes, obesity, elevated LDL-C, and atrial fibrillation as compared to men. Similar results were found in a study that included stroke patients aged 75 years or older from 2009 to 2011 in China [30]. A study from Korea showed a higher prevalence of diabetes in men under the age of 65 years, but diabetes was more prevalent in women over 65 years of age [31]. Atrial fibrillation has been reported as more common in female stroke patients [32], and women suffer more cardioembolic strokes as a result. In the present study, men were far more likely to drink and smoke than women, which is consistent with the findings of the Ludwigshafen Stroke Study [33]. Roquer et al. [34] also found that alcohol overuse and smoking are more common among males. Unlike the above studies [30–34], no sex difference in hypertension was observed in our population, which is consistent with a single prospective study [35].

We found risk factors in stroke survivors varied by age. Stroke survivors aged 60–69 years had higher proportions of smoking, alcohol consumption, diabetes, overweight or obese patients, and elevated LDL-C than those aged 70 or older, which is consistent with findings from Israel [36] and western Norway [37]. Higher mortality in older patients, especially in relation to the aforementioned risk factors, might explain lower risk factor prevalence among older patients [38]. Another possible explanation in China is that patients in this particular age group historically have spent most of their lives in relatively deprived conditions, reducing the chance that they have been exposed to risk factors for diabetes, obesity, and dyslipidemia.

Diabetes and atrial fibrillation were more prevalent in urban than in rural stroke survivors, while smoking and alcohol consumption were more common in rural stroke survivors; these findings are consistent with those of Liu et al [39]. Urban patients consume more calories than rural patients [40], which may lead to a higher prevalence of diabetes. Poor knowledge of stroke risk factors in rural areas [41] may lead to unwillingness to change unhealthy lifestyle habits such as smoking and alcohol consumption.

Geographically, stroke risk factors also varied between provinces. The northwestern province Ningxia had the highest prevalence of atrial fibrillation and a high prevalence of elevated LDL-C, physical inactivity, and overweight or obese patients, which may explain its high stroke prevalence.

## Limitations

Several potential limitations of this study should be noted. First, the participants were interviewed by researchers in community hospitals. Those who suffered from serious disability were unable to participate in this study, which means that the stroke prevalence in our study might be underestimated. Second, stroke subtypes were not analyzed; thus, we were unable to accurately identify different characteristics among stroke subtypes. Third, socio-economic analysis is a provincial comparison that may be biased by geographical factors.

## Conclusions

In conclusion, stroke prevalence in China is in line with median worldwide stroke prevalence. Traditional risk factors remain highly prevalent in stroke survivors, among which hypertension was the most common. Stroke prevalence rates and risk factors varied by age, sex, and sociogeological factors. In 2011, the Ministry of Health began the National Stroke Screening Survey. The Survey has established Stroke Intervention Base Hospitals, which bear the responsibility not only to provide standardized therapies and prevention, but also to educate the public about strokes. Targeted interventions that aim to control stroke risk factors and increased public education may help reduce stroke burden and recurrence rates.

## Electronic supplementary material

Below is the link to the electronic supplementary material.

Supplementary material 1 (DOCX 14 KB)

Supplementary material 2 (DOCX 17 KB)

Supplementary material 3 (DOCX 18 KB)

Supplementary material 4 (JPG 113 KB)
