# Supplementary material for: Prevalence and risk factors of stroke in the elderly in Northern China: data from the National Stroke Screening Survey
Source: J Neurol. 2019 Apr 15;266(6):1449–58. doi: 10.1007/s00415-019-09281-5 (PMC6517347; doi:10.1007/s00415-019-09281-5)
Supplement: Supplementary file 1 — Supplementary material 1 (DOCX 14 KB) [file 415_2019_9281_MOESM1_ESM.docx]

**Supplemental Table 1 Baseline population characteristics (N=144,722)**

|  | Total |
| --- | --- |
|  | N (%) |
| Gender |  |
| Male | 65432 (45.21) |
| Female | 79290 (54.79) |
| Age(years) |  |
| 60-69 | 88108 (60.88) |
| 70-79 | 43688 (30.19) |
| ≥80 | 12926 (8.93) |
| Provinces |  |
| Beijing | 12255 (8.47) |
| Tianjin | 10129 (7.00) |
| Hebei | 10563 (7.30) |
| Shanxi | 10971 (7.58) |
| Inner Mongolia | 4165 (2.88) |
| Liaoning | 8287 (5.73) |
| Jilin | 5420 (3.75) |
| Heilongjiang | 4383 (3.03) |
| Shandong | 32031 (22.13) |
| Henan | 20532 (14.19) |
| Shanxi | 9431 (6.52) |
| Gansu | 7988 (5.52) |
| Ningxia | 2765 (1.91) |
| Xinjiang | 5802 (4.01) |
| Residents |  |
| Urban | 67077 (46.35) |
| Rural | 77645 (53.65) |
