# Supplementary material for: Prevalence and risk factors of stroke in the elderly in Northern China: data from the National Stroke Screening Survey
Source: J Neurol. 2019 Apr 15;266(6):1449–58. doi: 10.1007/s00415-019-09281-5 (PMC6517347; doi:10.1007/s00415-019-09281-5)
Supplement: Supplementary file 2 — Supplementary material 2 (DOCX 17 KB) [file 415_2019_9281_MOESM2_ESM.docx]

**Supplemental Table 2 Recurrent stroke risk in stroke survivors stratified by ESRS**

|  | Total | 0-2 | 3-9 | *P* |
| --- | --- | --- | --- | --- |
|  | n（%） | n(%) | n（%） |  |
| Total | 7078 (100.00) | 2202 (31.11) | 4876 (68.89) |  |
| Gender |  |  |  | <0.001 |
| Male | 3709 (100.00) | 987 (26.61) | 2722 (73.39) |  |
| Female | 3369 (100.00) | 1215 (36.06) | 2154 (63.94) |  |
| Age |  |  |  |  |
| 60-69 | 4113 (100.00) | 1733 (42.13) | 2380 (57.87) | <0.001 |
| 70-79 | 2402 (100.00) | 352 (14.65) | 2050 (85.35) |  |
| ≥80 | 563 (100.00) | 117 (20.78) | 446 (79.22) |  |
