# Supplementary material for: Prevalence and risk factors of stroke in the elderly in Northern China: data from the National Stroke Screening Survey
Source: J Neurol. 2019 Apr 15;266(6):1449–58. doi: 10.1007/s00415-019-09281-5 (PMC6517347; doi:10.1007/s00415-019-09281-5)
Supplement: Supplementary file 3 — Supplementary material 3 (DOCX 18 KB) [file 415_2019_9281_MOESM3_ESM.docx]

**Supplemental Table 3 Main risk factors of stroke in stroke survivors by province**

|  | Smoking | Alcohol consumption | Physical inactivity | Hypertension | Diabetes | Overweight or obese | Elevated LDL-C | Atrial fibrillation |
| --- | --- | --- | --- | --- | --- | --- | --- | --- |
|  | % | % | % | % | % | % | % | % |
| **Total** | 27.26 | 15.45 | 50.01 | 80.18 | 23.24 | 38.14 | 73.36 | 8.80 |
| **Provinces** |  |  |  |  |  |  |  |  |
| Beijing | 28.91 | 16.47 | 56.13 | 83.72 | 32.44 | 40.46 | 77.48 | 7.57 |
| Tianjin | 41.25 | 18.83 | 58.32 | 82.51 | 26.13 | 44.06 | 70.41 | 9.05 |
| Jilin | 34.92 | 19.84 | 31.75 | 83.20 | 19.25 | 28.24 | 70.37 | 12.10 |
| Liaoning | 25.10 | 10.31 | 44.16 | 72.71 | 19.10 | 30.52 | 76.80 | 8.25 |
| Heilongjiang | 20.28 | 11.74 | 38.08 | 65.96 | 16.25 | 29.37 | 67.14 | 8.36 |
| Inner Mongolia | 20.75 | 18.40 | 83.02 | 80.66 | 41.98 | 31.13 | 85.48 | 8.02 |
| Shandong | 33.68 | 22.76 | 38.19 | 83.77 | 18.36 | 44.84 | 80.72 | 10.24 |
| Ningxia | 20.34 | 7.91 | 61.58 | 83.62 | 19.77 | 44.63 | 84.18 | 28.81 |
| Shanxi | 21.07 | 9.87 | 55.20 | 73.60 | 22.54 | 30.37 | 67.61 | 4.71 |
| Shaanxi | 25.41 | 8.84 | 49.72 | 59.09 | 21.82 | 13.59 | 61.24 | 4.59 |
| Hebei | 25.16 | 16.46 | 67.41 | 84.02 | 26.42 | 43.53 | 65.98 | 8.52 |
| Xinjiang | 15.92 | 10.83 | 43.31 | 76.43 | 31.85 | 44.94 | 64.97 | 8.86 |
| Henan | 18.38 | 7.04 | 49.52 | 85.34 | 25.51 | 41.41 | 70.24 | 4.37 |
| Gansu | 26.87 | 16.00 | 50.25 | 82.81 | 25.52 | 30.92 | 68.23 | 14.79 |
