# Supplementary figures and images for: Prevalence and risk factors of stroke in the elderly in Northern China: data from the National Stroke Screening Survey
Source: J Neurol. 2019 Apr 15;266(6):1449–58. doi: 10.1007/s00415-019-09281-5 (PMC6517347; doi:10.1007/s00415-019-09281-5)

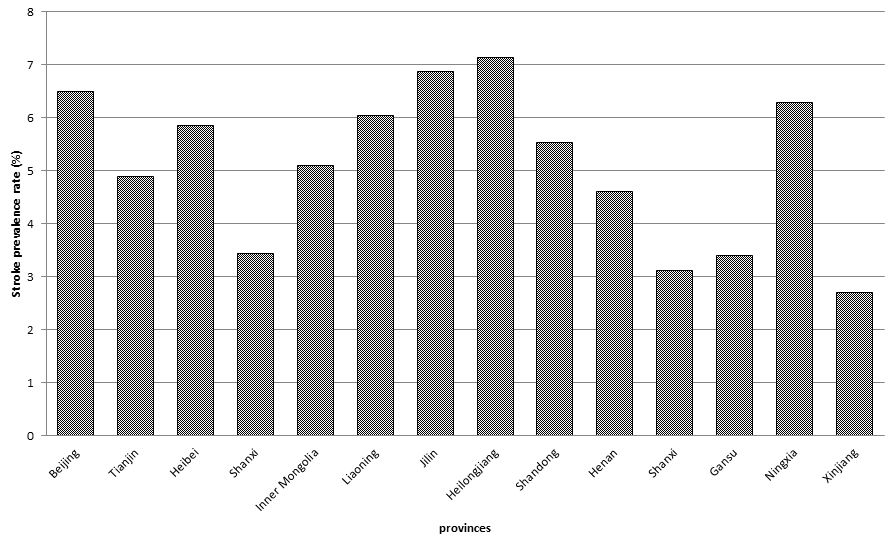

Supplement: Supplementary file 4 — Supplementary material 4 (JPG 113 KB) [file 415_2019_9281_MOESM4_ESM.jpg]
